# Effects of LC-PUFA Supplementation in Patients with Phenylketonuria: A Systematic Review of Controlled Trials

**DOI:** 10.3390/nu11071537

**Published:** 2019-07-06

**Authors:** María Luz Couce, María José de Castro, Carmela de Lamas, Rosaura Leis

**Affiliations:** 1 Department of Pediatrics, University Clinical Hospital of Santiago de Compostela, 15706 Santiago de Compostela, Spain; 2 IDIS-Health Research Institute of Santiago de Compostela, 15706 Santiago de Compostela, Spain; 3 CIBERER, Pabellón 11, 28029 Madrid, Spain; 4 Universidade de Santiago de Compostela, 15704 Santiago de Compostela, Spain; 5 Department of Pediatrics, Pediatric Metabolism and Research Unit, Reina Sofia University Hospital, IMIBIC, 14004 Cordoba, Spain

**Keywords:** arachidonic acid, cognitive function, docosahexaenoic acid, long-chain polyunsaturated fatty acids, phenylketonuria, visual function

## Abstract

Evidence suggests a role of long chain polyunsaturated fatty acids (LC-PUFA), in which animal foods are especially rich, in optimal neural development. The LC-PUFAs docosahexaenoic acid (DHA) and arachidonic acid, found in high concentrations in the brain and retina, have potential beneficial effects on cognition, and motor and visual functions. Phenylketonuria (PKU) is the most common inborn error of amino acid metabolism. The treatment of PKU consists of a phenylalanine-free diet, which limits the intake of natural proteins of high biological value. In this systematic review, we summarize the available evidence supporting a role for LC-PUFA supplementation as an effective means of increasing LC-PUFA levels and improving visual and neurocognitive functions in PKU patients. Data from controlled trials of children and adults (up to 47 years of age) were obtained by searching the MEDLINE and SCOPUS databases following Preferred Reporting Items for Systematic Reviews and Meta-Analyses (PRISMA) guidelines. For each selected study, the risk of bias was assessed applying the methodology of the Cochrane Collaboration. The findings indicate that DHA supplementation in PKU patients from 2 weeks to 47 years of age improves DHA status and decreases visual evoked potential P100 wave latency in PKU children from 1 to 11 years old. Neurocognitive data are inconclusive.

## 1. Introduction

Phenylketonuria (PKU; OMIM 261600) is an inborn error of phenylalanine (Phe) metabolism caused by an inherited deficiency in L-phenylalanine-4-hydroxylase (PAH; EC 1.14.16.1) activity, leading to elevated levels of Phe in body fluids [1]. Of patients with high phenylalanine concentrations, 98% have a defect in PAH and 1–2% in tetrahydrobiopterin metabolism. Children with PKU diagnosed by newborn screening who begin dietary treatment during the neonatal period usually show normal neurological development [2,3]. However, these patients may have lower intelligence quotients [4] and exhibit mild neuropsychological disturbances including impaired motor skills, visual function, attention, inhibition, and memory [5,6], especially when compared with non-phenylketonuric siblings [7] and healthy individuals [8,9]. PKU treatment consists of lifelong restriction of Phe intake by limiting the amount of natural protein in the diet, combined with administration of a Phe-free amino-acid mixture [10]. More recently, a synthetic form of tetrahydrobiopterin (6R-BH4) has been used to treat selected patients who have moderate forms of PKU and respond to the BH4 loading test [11,12]. Owing to a tendency to exclude protein-rich animal food from their diet, micronutrient deficiencies are common in PKU patients [13,14,15].

Meat and fish are the main sources of long-chain polyunsaturated fatty acids (LC-PUFA) in humans, and although they are produced endogenously, dietary intake is the key determinant of LC-PUFA levels [16]. Docosahexaenoic acid (DHA) and arachidonic acid (AA) are the most important LC-PUFAs of the *n*-3 and *n*-6 series, respectively [17]. Both are structural components of cell membranes and influence their biological functions, including enzymatic activity, transport through ion channels, and signal transduction [18], especially in the nervous system and the retina [19]. Incorporation of DHA and AA in these tissues during the pre- and postnatal periods has been correlated with visual, cognitive, and motor functions in humans [20,21,22].

The low-Phe diet of PKU patients has been linked to insufficient blood levels of LC-PUFAs, which may contribute to the mild neurological, cognitive, and visual alterations described in these patients [23]. However, to date no conclusive evidence supports a link between the PKU diet, the LC-PUFA profile and the clinical status of PKU patients [24]. In this systematic review, we present a comprehensive overview of evidence from clinical trials assessing a correlation between the PKU diet, LC-PUFA status, and neurocognitive and visual functions.

## 2. Methods

This review was conducted following the guidelines for Preferred Reporting Items for Systematic Reviews and Meta-Analyses (PRISMA) [25] and was registered in the International Prospective Register of Systematic Reviews (PROSPERO) with the number CRD42019133315. The review question, which was formulated following the PICOS (Population, Intervention, Comparison, Outcomes and Settings) criteria [26] (Table 1), was as follows: Does LC-PUFA supplementation influence visual and neurocognitive functions in patients with phenylketonuria?

### 2.1. Inclusion and Exclusion Criteria

Studies were selected applying the following inclusion criteria: all controlled studies, randomized or not, of patients with PKU of any age and ethnicity that were published between 1 January 1995 and 1 April 2019. Studies in which LC-PUFA supplementation was administered parenterally and those lacking a control group that did not receive LC-PUFA supplementation were excluded.

### 2.2. Intervention Types

Studies were not restricted according to the duration of supplementation or the type or dose of LC-PUFAs administered. All studies in which patients received oral LC-PUFA supplementation and the effects were compared with a non-supplemented group were considered for inclusion.

### 2.3. Primary Outcome Measures

Visual evoked potential (VEP) variables, specifically alterations in P100 wave and P1 peak latencies (in ms), were the primary outcome measures used to assess the effects of LC-PUFA supplementation on visual function. For the assessment of neurocognitive function, data from any study that included some form of evaluation of psychomotor development were considered. Circulating and erythrocyte lipid levels and changes in lipid levels (mg/L, mmol/L, or % change) after supplementation were considered valid measures for the assessment of effects on lipid status.

### 2.4. Literature Search

The PUBMED and SCOPUS databases were searched using the MeSH terms “Fatty Acids, Unsaturated” and “Phenylketonurias”. “Fatty Acids, Unsaturated” (Mesh Terms) AND “Phenylketonurias” (Mesh Terms) was the search strategy used in PUBMED. SCOPUS was searched using the following formula: “Fatty acids” AND “Phenylketonurias”, excluding results from animal studies.

### 2.5. Study Selection

Two authors (MJDC and CDL) independently selected studies from the 33 articles reviewed in full. In cases in which there was a lack of consensus regarding selection, discrepancies were arbitrated by MLC and RL. Nine articles [27,28,29,30,31,32,33,34,35] were ultimately selected for inclusion in the review.

### 2.6. Data Extraction

The following data were extracted from each study: publication year; number of participants by sex; age; study type; intervention characteristics. Data on study duration, outcomes, results, and conclusions were separately extracted by two investigators. Any discrepancies in opinions were arbitrated by MLC and RL.

### 2.7. Assessment of Risk of Bias

Following the methodology of the Cochrane Collaboration, London, UK [36], two evaluators independently studied the risks of bias. The articles were analyzed individually, and the corresponding risk of bias was classified as high, uncertain, or low depending on the risk of selection bias (random sequence generation, allocation concealment), performance bias (blinding of participants and personnel), detection bias (blinding of outcome assessment), attrition bias (incomplete outcome data), reporting bias (selective reporting), and other forms of bias. A third and a fourth reviewer arbitrated in cases of discrepancies of opinion. We also evaluated the general risk of bias in the group of articles included in the systematic review, expressed as a percentage of articles that present a risk of specific bias in relation to the total number of studies included.

## 3. Results

**Figure 1:**
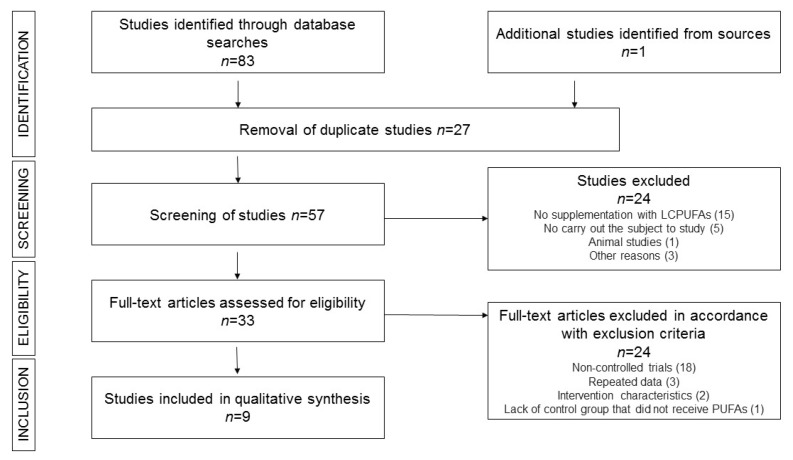
Flow chart depicting the literature search process.

The results of each step of the bibliographic search are shown in Figure 1. Of the 84 results from the first search (PUBMED, 53; SCOPUS, 30; other sources, 1), 27 duplicate articles were excluded. Twenty-four articles were excluded after revision of the abstract. Of the 33 articles considered for evaluation of the complete text, nine articles were ultimately included in the systematic review [27,28,29,30,31,32,33,34,35].

Table 2, Table 3 and Table 4 show the main characteristics of the selected clinical trials. Publication dates range from 1995 to 2017. Only one [35] of the nine articles included was published before the year 2000. The combined study population of the nine clinical trials included in this review was 419 individuals, 365 (87.1%) of whom had phenylketonuria. Only two studies included a healthy control group [29,33] and the remaining seven, a PKU control group [27,28,30,31,32,34,35]. Ages ranged from 2.1 weeks to 47 years. The mean sample size was 46 ± 27 participants (range, 20–109). The median intervention period was 6 months (range, 3–12 months). Supplementation consisted of a phenylalanine-free LC-PUFA-supplemented infant formula [30,31,32] or DHA [27,28] or fish-oil [29,33,34,35] capsules. In one study [32], no information on the DHA supplementation dose was provided. In all other studies, the DHA dose ranged from 0.1 to 15 mg/kg/day. In four studies, supplementation consisted of a combination of omega 6 and omega 3 [30,31,32,34], with omega 6 to omega 3 ratios ranging from 1:1 to 3:1.

### 3.1. LC-PUFA Supplementation and Circulating and Erythrocyte Lipids

Changes in circulating and erythrocyte lipids were evaluated in seven of the nine studies [27,28,30,31,32,34,35] (Table 2), all of which reported significant differences in DHA levels in LC-PUFA-supplemented groups versus controls. Of those seven trials, five [27,28,32,34,35] reported higher levels of DHA in the LC-PUFA-supplemented group, while two trials concerning LC-PUFA supplementation in infants [30,31] reported a significantly lower decrease in DHA levels compared with controls. Four of the articles [27,28,30,35] evaluated DHA levels in plasma and four [28,31,32,34] in erythrocyte lipids. Only one study [28] evaluated DHA levels in both plasma and erythrocyte lipids. Of the two studies in which cholesterol and triglyceride levels were measured [27,35], the study by Demmelmair et al., in which patients received the lower DHA dose [27], reported no differences in these variables between groups, while the other [35] reported significantly lower triglyceride levels in the LC-PUFA-supplemented group.

### 3.2. LC-PUFA Supplementation and Visual Function

Four of the studies included in our review provided VEP data [27,31,33,34] (Table 3). All measured P100 wave latency (Pattern VEP), and one [31] also measured the P1 peak (Flash VEP). Two of the four studies in which the highest dose (15 mg/kg) of DHA [33,34] was administered reported significant decreases in P100 wave latency in the supplementation group, which received DHA capsules in both cases. The two remaining studies [27,31], one [31] in which newborns were supplemented with formula and the other one [27] with 0.1–7 mg/kg of DHA supplementation, reported no significant differences in visual function between groups.

### 3.3. LC-PUFA Supplementation and Neurocognitive Function

Neurocognitive function was evaluated in four studies [27,28,29,31] (Table 4): three studies [27,28,31] assessed the cognitive area and two studies [27,29] the motor function. Only one [29] reported significant differences in psychomotor development between supplemented and non-supplemented groups. There was considerable variability among these four studies in terms of age and outcomes. Ages ranged from 20 ± 6.9 weeks in the study by Agostoni et al. [31] to 12–47 years in the study by Yin et al. [28].

There was also considerable heterogeneity regarding the scales used to evaluate cognition: one study [28] assessed verbal ability using the Peabody picture vocabulary test, executive function using the Delis Kaplan executive function system and processing speed using the Woodcock–Johnson III tests of cognitive ability and achievement; another [27] calculated the intellectual quotient using Raven’s progressive matrices; and a third study [31] assessed the coefficient of development using the Bayley test. The two studies that evaluated motor function [27,29] both used the Rostock–Oseretzky scale.

### 3.4. Risk-of-Bias Assessment

For all studies included in our review, we concluded that there was a low risk of selection bias (allocation concealment) and an unclear risk of reporting bias (selective reporting). The percentage of studies for which the risk of different forms of bias was considered low was as follows: attrition bias (incomplete outcome data), 89%; selection bias (random sequence generation), 66%; performance bias (blinding of participants and personnel), 66%; detection bias (blinding of outcome assessment), 55%. We concluded that there was a risk of other forms of bias in 55% of studies, due to a lack of standardized protocols in three multicenter studies [27,31,32] and the lack of a control group composed of non-LC-PUFA-supplemented PKU patients in two studies [29,33].

The study for which the risk of biased results was greatest was that of Cleary et al. [32]; a multicenter study for which no standardized protocol was described. The risk of attrition bias was also high for this study, given the omission of an intention-to-treat analysis. The risk of bias was lowest for the studies by Koletzko et al. [29] and Yin et al. [28].

Additional information on the risk-of-bias analysis (risk-of-bias graph and summary) is provided in the Supplementary Materials Figures S1 and S2.

## 4. Discussion

This systematic review of controlled trials regarding LC-PUFA supplementation in children and adults with PKU reveals that the addition of DHA at doses ≥10 mg/kg/day to the patient’s Phe-restricted diet decreases VEP latencies. However, no conclusive evidence supports a relationship between LC-PUFA supplementation and neurocognitive outcomes in these patients.

DHA and AA are the most important LC-PUFAs of the *n*-3 and *n*-6 series, respectively. These key structural components of neuronal cell membranes are of crucial importance for brain development and retinal function [37]. In randomized clinical trials, LC-PUFA supplementation is associated with improved visual and cognitive maturation in full-term and, in particular, preterm infants [38,39,40]. These outcomes in preterm infants may be linked to the greater predisposition of these children to LC-PUFA deficiency due to fetal accretion of DHA (which usually occurs during the third trimester), an inability to convert precursor fatty acids to DHA, and low postnatal DHA intake [41].

PKU patients are another population at risk of LC-PUFA deficiency; the typical Phe-restricted diets of these patients provide low amounts of animal products, which are the main source of LC-PUFAs [42,43]. Moreover, excess Phe is catabolized to phenylpyruvate and phenyllactate, which are reported to inhibit endogenous synthesis of DHA and AA [44]. A 2013 systematic review and meta-analysis of nine case control studies and six randomized controlled trials concluded that PKU patients have significantly lower levels of both DHA and AA in all biomarkers studied than healthy controls [24]. In line with this suboptimal LC-PUFA status in PKU patients, studies of children with amino acid metabolism disorders have described reduced LC-PUFA intake (a consequence of dietary protein restriction) and lower plasma and erythrocyte membrane concentrations of DHA than healthy controls [45,46,47]. The results of studies of AA status in these patients are inconclusive (ranging from normal to reduced), suggesting that endogenous synthesis may be sufficient to ensure adequate AA status in some cases [48].

The findings of this systematic review indicate that DHA supplementation in PKU patients significantly increases DHA levels in plasma and/or erythrocyte membranes [27,28,29,30,31,32,33,34,35]. It should be noted that erythrocyte fatty acid composition yields more information regarding long-term LC-PUFA status and is less influenced by fasting, appearing to be a more valuable biomarker [49,50]. While the most commonly administered dose of DHA was 10–15 mg/kg/day, significant increases in DHA levels were observed even with lower doses (0.1–7 mg/kg/day) [27]. There are insufficient data to define an optimal LC-PUFA supplementation dose for PKU patients of different age groups, and optimal DHA intake in infants and children remains a topic of debate according to both the ESPGHAN Committee on Nutrition and The European Food Safety Authority Panel on Dietetic Products, Nutrition and Allergies (NDA). Despite the lack of appropriate data on which to base dietary reference values for pediatric patients, the aforementioned organizations have proposed an intake of 100 mg/day for patients aged 6–24 months and 250 mg/day for those aged 2 years and older [51].

VEP testing was conducted to assess central nervous system (CNS) function in four of the studies included in this review [27,31,33,34]. VEP is widely used in studies of neural maturation as it provides a sensitive means of assessing the function of a major CNS pathway. P100 wave latency is considered the most reliable clinical indicator, as is the variable least affected by technical factors and the degree of patient cooperation [52]. Longer VEP latencies, which are observed in PKU patients not receiving LC-PUFA supplementation [52], indicate a lower speed of information processing from the retina to the visual cortex. It should be noted that the controlled trials (CTs) and randomized controlled trials (RCTs) in which shorter P100 wave latencies were observed after intervention were those in which the patients received higher doses of DHA (10–15 mg/kg/day) [33,34]. In the two RCTs [27,31] that reported no differences in VEP latencies after LC-PUFA supplementation, patients received lower (0.1–7 mg/kg/day) or uncontrolled doses (i.e., a LC-PUFA-supplemented, Phe-free formula) of DHA. In the latter study [31], higher levels of DHA in erythrocyte membranes were significantly correlated with a shorter P100 wave latency after adjustment for age. This observation suggests that the LC-PUFA intake of these patients was irregular and, in many cases, insufficient to alter the clinical outcome.

None of the studies included in this review specifically evaluated retinal function. However, a 2013 study [53] assessed visual function in PKU patients using a comprehensive ophthalmological test battery. Electroretinography (ERG), which allows for objective measurement of retinal function, revealed that PKU patients showed abnormalities in scotopic and photopic ERG amplitudes and latencies not observed in healthy individuals. It should be noted that this pattern of ERG alterations has also been described in animal models of LC-PUFA depletion and is likely related to abnormal DHA metabolism in photoreceptor membranes [54,55,56].

Evidence suggests that LC-PUFA supplementation may improve neurocognitive function, including motor skills [29], in PKU patients. Children with early-treated PKU can present structural alterations in cerebral white matter myelin [57,58,59] that may be associated with high Phe levels, but also with low DHA concentrations. However, because beneficial effects were reported in only one CT [29], and given the considerable variability across the studies included in this review in terms of the dose used, form of supplementation, functional outcome measures, and neurocognitive scales used, the available evidence is inconclusive. Previous reviews that have assessed the effects of LC-PUFA supplementation on cognitive performance in children and adults without PKU [60,61,62,63] have reported similarly inconclusive findings, in large part due to the marked heterogeneity in the interventions and outcome measures used.

The duration of the intervention is another important variable to consider when examining the functional effects of LC-PUFA supplementation. In their study of the effects of DHA administration in pediatric PKU patients and healthy controls, Agostoni et al. [64] found that P100 wave latencies and DHA status, both of which had improved in the PKU group during the intervention, returned to baseline levels 3 years after treatment discontinuation.

When evaluating the risk of bias for each of the studies included in this systematic review, not all forms of bias should be considered equally important. For example, because LC-PUFA levels and VEP latencies are objective measures, the selected studies are less likely to be affected by performance bias. Besides that, the reporting bias is unclear in all articles included, so the main forms of bias to consider in our review are selection bias, attrition bias, and detection bias.

Future studies should consider using standardized neurocognitive assessment scales and doses and durations of DHA supplementation in order to determine the tissue levels of DHA necessary to achieve significant homogenous clinical improvements. Moreover, data on the clinical course of patients who discontinue DHA supplementation could be particularly valuable, since the effects of DHA may disappear after discontinuation. Specifically, adjustment of these data for age would enable the identification of the most vulnerable stages of life and the optimum window of opportunity for intervention.

## 5. Conclusions

The results of this systematic review support the beneficial effects of DHA supplementation in PKU patients: deficient LC-PUFA status is corrected in patients from 2 weeks to 47 years of age, and P100 wave latency improves in children from 1 to 11 years old. However, evidence is inconclusive regarding the effect of DHA on neurocognitive function. Further research will be required to establish the optimal DHA dose and duration of intervention.

## Figures and Tables

**Table 1:** PICOS (Population, Intervention, Comparison, Outcomes and Settings) criteria [26] for the inclusion of studies evaluating the effects of long-chain polyunsaturated fatty acids (LC-PUFA) supplementation in phenylketonuria patients.

Parameter ^1^	Inclusion Criteria
Population	Patients with phenylketonuria
Intervention	Controlled LC-PUFA intake
Comparison	Non-exposed control group
Outcome	Visual and neurocognitive functions and fatty acid levels
Setting	Controlled trials

^1^ PICOS criteria [26].

**Table 2:** Effects of LC-PUFA supplementation on circulating and erythrocyte lipids in 299 phenylketonuria patients in controlled trials.

Reference	*n*	Age ^1^	Intervention	Trial Type (Duration of Intervention)	Outcome Measure	Results ^2^	Conclusion
Demmelmair et al. (2018) [27]	109	5–13 years	DHA capsules (IG1, 0.1–1.8 mg/kg/day; IG2, 1.9–7 mg/kg/day)	RCT (6 months)	Change in plasma lipid concentration	DHA (mg/L): IG1, 5.1 ± 10.3; IG2, 3.19.5 ± 13.6; CG, 0.0 ± 9.1	Significant increase in DHA levels
						TC (mmol/L): IG1, 0.0 ± 0.5; IG2, 0.1 ± 0.5; CG, −0.1 ± 0.6	
						HDL (mmol/L): IG1, 0.0 ± 0.3; IG2, 0.0 ± 0.2; CG, −0.0 ± 0.3	
						LDL (mmol/L): IG1, 0.2 ± 0.5; IG2, 0.1 ± 0.5; CG, −0.3 ± 1.2	
						TG (mmol/L): IG1, 0.0 ± 0.5; IG2, −0.1 ± 0.5; CG, −0.1 ± 0.6	
Yi et al. (2011) [28]	33 (33 F)	12–47 years	DHA capsules (10 mg/kg/day)	RCT (4.5 months)	Plasma and erythrocyte FA	DHA (weight % FA): IG, 3.14 ± 0.57; CG, 0.97 ± 0.34	Significant increase in plasma DHA and erythrocyte FA levels
						Erythrocyte DHA (weight % erythrocyte FA): IG, 5.82 ± 1.26; CG, 2.35 ± 0.78	
Koletzko et al. (2007) [30]	21 (8 F)	2.1 ± 0.9 weeks	Test-treatment formula (DHA, 0.23 g/100 g FA.	RCT (12 months)	Plasma phospholipid FA	DHA (weight % FA): IG, 3.08 ± 0.1; CG, 1.52 ± 0.19	Significant less decline of DHA levels
			Omega 6: omega 3 ratio, 2:1)				
Agostoni et al. (2006) [31]	42 (22 F)	20 ± 6.9 weeks	Test-treatment formula (DHA, 0.3 g/100 g FA.	RCT (12 months)	Median change in LC-PUFA concentration in erythrocyte MB phospholipids	% change DHA:	Significant less decline of DHA levels
						IG, −22%; CG, −64%	
						% change AA:	
			Omega 6: omega 3 ratio, 2.5:1				
						IG, –5%; CG, –19%	
Cleary et al. (2006) [32]	53	1–10 years	Test-treatment formula (PUFA, 2.8 g/100 g.	RCT (20 weeks)	Median change in LC-PUFA concentration in erythrocyte MB phospholipids	% change DHA: IG, +19%; CG, +0.5%	Significant increase in DHA levels
			Omega 6: omega 3 ratio, 3:1)			% change AA: IG, +0.5%; CG, +7.6%	
Agostoni et al. (2000) [34]	20 (9 F)	10.7 ± 2.4 years (IG)	Fish oil capsules (DHA, 15 mg/kg/day.	RCT (12 months)	LC-PUFA concentration in erythrocyte lipids	Erythrocyte PC (weight % FA): EPA: IG, 0.1 ± 0.07; CG, 0.1 ± 0.04	Significant increase of DHA levels
						DHA: IG, 0.9 ± 0.3; CG, 0.4 ± 0.2. AA: IG, 5.39 ± 1.16; CG, 5.83 ± 0.98	
			AA:DHA ratio, 1:1				
		10.5 ± 2.8 years (CG)					
						Erythrocyte PEA (weight % FA): EPA: IG, 0.3 ± 0.1; CG, 0.2 ± 0.1 DHA: IG, 3.7 ± 1.7; CG, 1.3 ± 0.9. AA: IG, 16.1 ± 5.2; CG, 14.5 ± 7.3	
Agostoni et al. (1995) [35]	21	5–10 years	Fish oil capsules (DHA, 15 mg/kg/day; EPA, 22.5 mg/kg/day)	RCT (6 months)	Plasma lipid concentration	TC (mmol/L): IG, 3.12 ± 0.67; CG, 3.41 ± 0.28	Significant decrease in triglycerides and increase in *n*-3 LC-PUFA levels
						HDL (mmol/L): IG, 1.06 ± 0.18; CG, 1.18 ± 0.23	
						LDL (mmol/L): IG, 1.75 ± 0.72; CG, 1.73 ± 0.49	
						TG (mmol/L): IG, 0.68 ± 0.16; CG, 1.09 ± 0.47	
						LC-PUFA (weight % FA). EPA: IG, 1.96 ± 0.79; CG, 0.27 ± 0.06	
						DHA: IG, 2.94 ± 0.88; CG, 0.73 ± 0.08; AA: IG, 5.39 ± 1.16; CG, 5.83 ± 0.98	

AA, arachidonic acid; CG, control group; CT, controlled trial; DHA, docosahexaenoic acid (22:6, *n*-3); EPA, eicosapentaenoic acid (20:5, *n*-3); F, female; FA, fatty acid; HDL, high density lipoprotein cholesterol; IG, intervention group; LC-PUFA, long chain polyunsaturated fatty acid; LDL, low-density lipoprotein cholesterol; MB, membrane; PC, phosphatidylcholine; PEA, phosphatidylethanolamine; RCT, randomized controlled trial; TC, total cholesterol;. TG, triglyceride. ^1^ Values (at entry) represent the range or the mean ± SD, as reported in the corresponding article. ^2^ Values represent the mean or mean ± SD, as reported in the corresponding article.

**Table 3:** Effects of LC-PUFA supplementation on visual function in 237 subjects in controlled trials.

Reference	*n*	Age ^1^	Intervention	Type and Duration of Intervention	Outcome Measure	Results ^2^	Conclusion
Demmelmair et al. (2018) [27]	109	5–13 years	DHA capsules (IG1, 0.1–1.8 mg/kg/day; IG2, 1.9–7 mg/kg/day)	RCT—6 months	Change in P100 wave latency (ms)	Pattern-reversal. 15: IG1, 0.5 ± 8.7; IG2, −0.6 ± 4.7; CG, 1.3 ± 3.5	No significant differences
Agostoni et al. (2006) [31]	42 (22 F)	20 ± 6.9 weeks	Test-treatment formula (DHA, 0.3 g/100 g FA.	RCT—12 months	P100 wave (pattern VEP) and P1 peak (flash VEP) latencies (ms)	Pattern-reversal: IG, 120 ± 24; CG, 107 ± 8	No significant differences
			Omega 6: omega 3 ratio, 2.5:1)			Flash: IG, 108 ± 15; CG, 115 ± 24	
Beblo et al. (2001) [33]	66 (34 F)	6.6 ± 1.5 years (CG)	Fish oil capsules (DHA, 15 mg/kg/day; EPA, 22.5 mg/kg/day)	CT—3 months	Change in P100 wave latency	No data	Significant decrease in P100 wave latency (5’, 15’)
				CG healthy children			
Agostoni et al. (2000) [34]	20 (9 F)	10.7 ± 2.4 years (IG)	Fish oil capsules (DHA, 15 mg/kg/day)	RCT—12 months	P100 wave latency (ms)	Pattern-reversal. 60’: IG, 104 ± 4; CG, 109 ± 9. 15’: IG, 107 ± 6; CG, 118 ± 11.	Significant decrease in P100 wave latency (15’, 2 Hz-1 J)
						Flash. 1 Hz-2 J: IG, 113 ± 10; CG, 114 ± 8.	
		10.5 ± 2.8 years (CG)					
			AA:DHA ratio, 1:1				
						2 Hz-1 J: IG, 111 ± 12; CG, 121 ± 8	

AA, arachidonic acid; CG, control group; CT, controlled trial; DHA, docosahexaenoic acid (22:6, *n*-3); F, female; EPA, eicosapentaenoic acid (20:5, *n*-3); FA, fatty acids; IG, intervention group; RCT, randomized controlled trial; VEP, visual evoked potentials. ^1^ Values (at entry) represent the range or mean ± SD, as reported in the corresponding article. ^2^ Values represent the mean ± SD, as reported in the corresponding article.

**Table 4:** Effects of LC-PUFA supplementation on neurocognitive function in 238 subjects in controlled trials.

Reference	*n*	Age ^1^	Intervention	Type and Time of Intervention	Outcome Measure	Results ^2^	Conclusion
Demmelmair et al. (2018) [27]	109	5–13 years	DHA capsules (IG1, 0.1–1.8 mg/kg/day; IG2, 1.9–7 mg/kg/day)	RCT—6 months	Changes in motometric Rostock–Oseretzky scale and Raven´s progressive matrices	Rostock–Oseretzky scale: IG1, 4.2 ± 6.3; IG2, 0.8 ± 9.1; CG, 2.9 ± 7.0	No significant differences
						Raven’s progressive matrices: IG1, 2.2 ± 15.8; IG2, 1.6 ± 13.8; CG, 9.5 ± 13.5	
Yi et al. (2011) [28]	33 (33 F)	12–47 years	DHA capsules (10 mg/kg/day)	RCT—4.5 months	Verbal ability (Peabody picture vocabulary test, third edition), executive function (Delis-Kaplan executive function system), and cognitive processing speed (Woodcock–Johnson III tests of cognitive ability and achievement)	Cognitive processing speed, factor score: IG, 98.8 ± 5.3; CG, 101 ± 5.4	No significant differences
						Cognitive inhibition: IG, 11.3 ± 1.5; CG, 11.4 ± 1.5	
						Cognitive flexibility: IG, 11.1 ± 1.4; CG, 10.8 ± 1.4	
Koletzko et al. (2009) [29]	54	6.3 ± 0.6 years	Fish oil capsules (DHA, 15 mg/kg/day; EPA, 22.5 mg/kg/day)	CT—3 months	Changes in motometric Rostock–Oseretzky scale	No data	Significant improvement in fine motor skills (especially coin sorting), dynamic balance, and total score in intervention group
				CG, healthy children			
Agostoni et al. (2006) [31]	42 (22 F)	20 ± 6.9 weeks	Test-treatment formula (DHA, 0.3 g/100 g FA.	RCT—12 months	Mental and psychomotor development (Bailey test, second edition)	Mental development: IG, 92.67 ± 16.02; CG, 93.19 ± 16.60	No significant differences
						Physical development: IG, 92 ± 13.32; CG, 97.69 ± 15.57	
			Omega 6: omega 3 ratio, 2.5:1)				

AA, arachidonic acid; CG, control group; CT, controlled trial; DHA, docosahexaenoic acid (22:6, *n*-3); EPA, eicosapentaenoic acid (20:5, *n*-3); F, female; FA, fatty acids; IG, intervention group; LC-PUFA, long chain polyunsaturated fatty acid; RCT, randomized controlled trial; VEP, visual evoked potentials. ^1^ Values (at entry) represent the range, mean, or mean ± SD, as reported in the corresponding article. ^2^ Values represent the mean ± SD, as reported in the corresponding article.

## Supplementary Materials

The following are available online at https://www.mdpi.com/2072-6643/11/7/1537/s1, Figure S1, Risk-of-bias summary: review of authors’ judgments on each risk-of-bias item for each study. Figure S2, Risk-of-bias graph: review of authors’ judgments on each risk-of-bias item, presented as percentages across studies.
