# Supplementary figures and images for: Effects of LC-PUFA Supplementation in Patients with Phenylketonuria: A Systematic Review of Controlled Trials
Source: Nutrients. 2019 Jul 6;11(7):1537. doi: 10.3390/nu11071537 (PMC6682937; doi:10.3390/nu11071537)

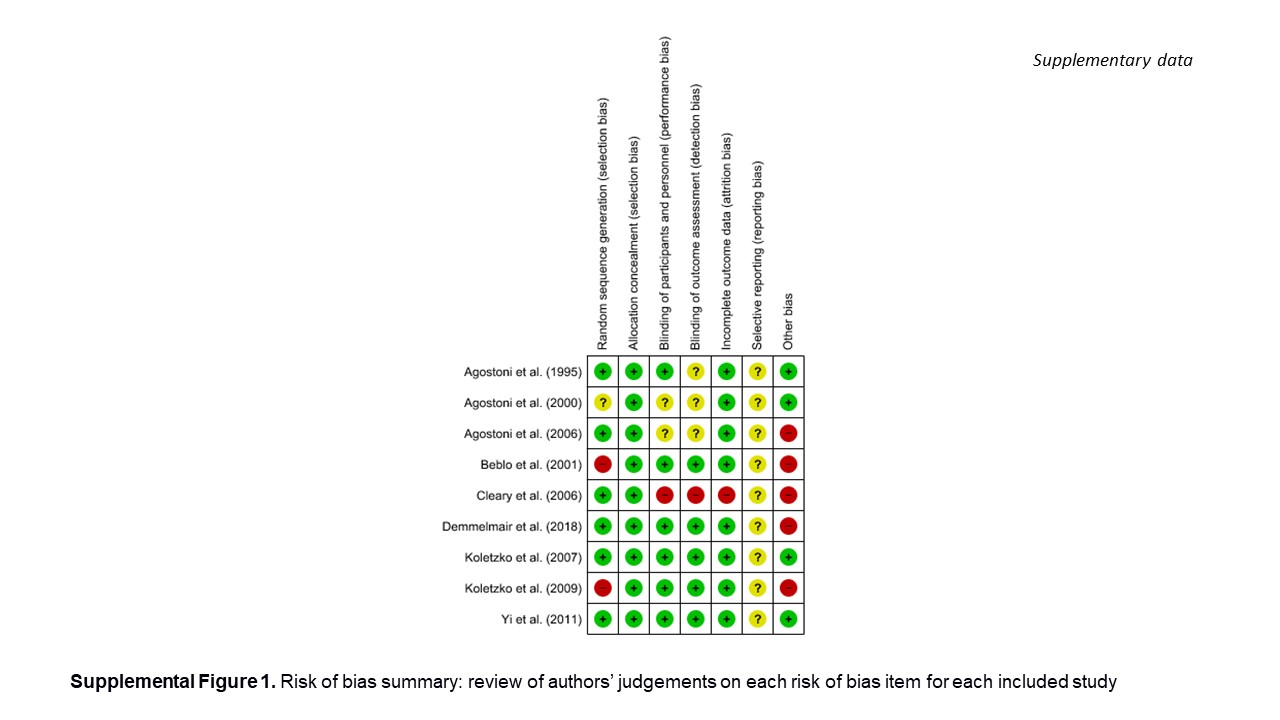

Supplement: Supplementary file 1 [file nutrients-11-01537-s001.zip › Supplemental figure 1.jpg]

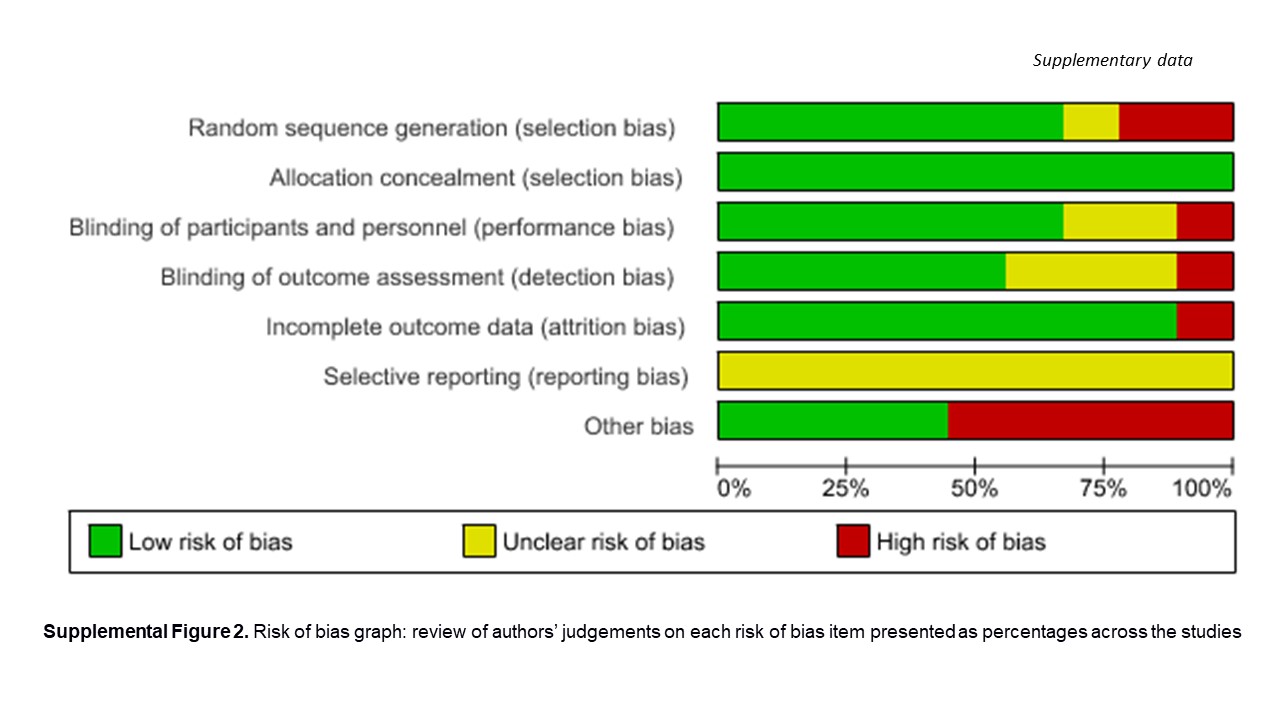

Supplement: Supplementary file 1 [file nutrients-11-01537-s001.zip › Supplemental figure 2.jpg]
